# Comparing two machine learning approaches in predicting lupus hospitalization using longitudinal data

**DOI:** 10.1038/s41598-022-20845-w

**Published:** 2022-09-30

**Authors:** Yijun Zhao, Dylan Smith, April Jorge

**Affiliations:** 1 Computer and Information Sciences Department, Fordham University, 113 W 60th St., New York, NY 10023 USA; 2 Division of Rheumatology, Allergy, and Immunology, Massachusetts General Hospital, Boston, USA; 3 Harvard Medical School, Boston, MA USA

**Keywords:** Systemic lupus erythematosus, Machine learning

## Abstract

Systemic lupus erythematosus (SLE) is a heterogeneous autoimmune disease characterized by flares ranging from mild to life-threatening. Severe flares and complications can require hospitalizations, which account for most of the direct costs of SLE care. This study investigates two machine learning approaches in predicting SLE hospitalizations using longitudinal data from 925 patients enrolled in a multicenter electronic health record (EHR)-based lupus cohort. Our first Differential approach accounts for the time dependencies in sequential data by introducing additional lagged variables between consecutive time steps. We next evaluate the performance of LSTM, a state-of-the-art deep learning model designed for time series. Our experimental results demonstrate that both methods can effectively predict lupus hospitalizations, but each has its strengths and limitations. Specifically, the Differential approach can be integrated into any non-temporal machine learning algorithms and is preferred for tasks with short observation periods. On the contrary, the LSTM model is desirable for studies utilizing long observation intervals attributing to its capability in capturing long-term dependencies embedded in the longitudinal data. Furthermore, the Differential approach has more options in handling class imbalance in the underlying data and delivers stable performance across different prognostic horizons. LSTM, on the other hand, demands more class-balanced training data and outperforms the Differential approach when there are sufficient positive samples facilitating model training. Capitalizing on our experimental results, we further study the optimal length of patient monitoring periods for different prediction horizons.

## Introduction

Systemic lupus erythematosus (SLE) is a chronic autoimmune disease characterized by heterogeneous disease manifestations, and disease activity can fluctuate over time. Patients with SLE can experience periods of severe disease flares, for which hospitalization may be necessary^1^. Hospitalizations for SLE are associated with significant morbidity and mortality and account for most of the direct costs of SLE care^2,3^. Predicting disease outcomes in chronic medical conditions such as lupus^4^ is challenging but critical to facilitate rigorous monitoring procedures and appropriate treatment. In recent years, data driven approaches such as machine learning (ML) models have have been applied to predicting clinical outcomes for SLE and other chronic conditions^5–9^. Clinical data associated with these studies are typically collected at regular time intervals and, thus, exhibit strong temporal dependencies. However, a common limitation is that most well-established models, including decision trees (DT)^10^, random forest (RF)^11^, logistic regression (LR)^12^, and neural networks (NN)^13^, are ill-suited for modeling time series data because they assume observations at different time steps are independent and identically distributed (i.i.d.).

**Figure 1:**
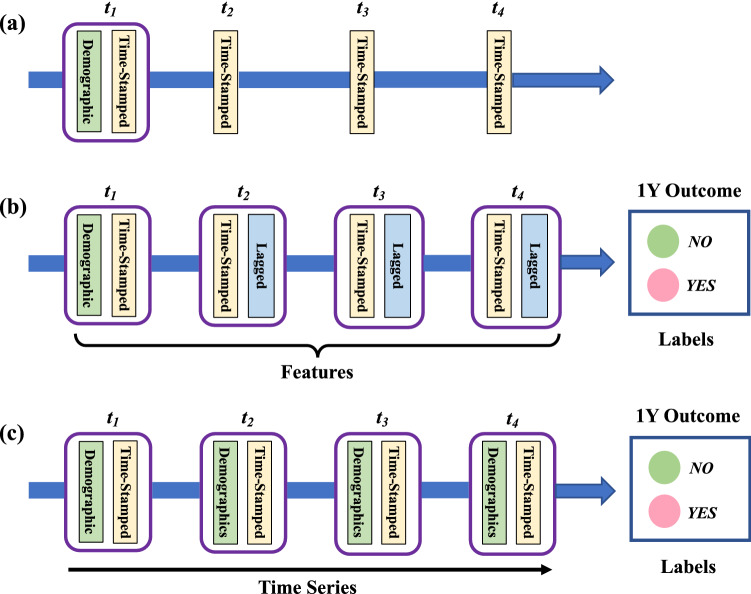
Experimental data construction. (**a**) Original patient longitudinal record extracted at regular time intervals. (**b**) Features and labels for the Differential approach. Additional lagged variables are created for subsequent time periods to capture the change of each clinical variable between the current and previous time periods. (**c**) Time series data for LSTM model. Demographic features are appended to the clinical features at each time step to form equal-length sequences to facilitate model training. Labels in both (**b**) and (**c**) indicate patients’ hospitalization outcomes in the year after the feature assessment periods.

An intuitive technique to capture data dependencies in non-temporal ML models is to focus on the changes in each temporal feature between consecutive observations in the longitudinal data, denoted as the “Differential” approach onward. Figure 1a illustrates the patient electronic health record (EHR) records acquired at regular intervals (e.g., 6M). In addition to static demographic features, each period contributes a set of time-stamped clinical features capturing a snapshot of the disease. Figure 1b presents the data construction process in the Differential approach for the task of predicting patients’ 1 year hospitalization outcomes using four observation periods (i.e., 2 years). Additional lagged variables are created for subsequent time periods to obtain the change of each clinical variable between the current and previous periods. Consequently, desired ML models can be trained using the entire panel of features, including patient demographics. Because dependencies in the longitudinal data are modeled with engineered lagged variables, the Differential approach facilitates the application of a rich family of non-temporal ML models (e.g., DT, RF, LR, NN, etc.) while still accounting for the progression of a patient’s disease. The feature engineering technique described here has delivered promising results in similar studies concerning the prediction of the disease courses in multiple sclerosis and lupus patients^9,14,15^ and in estimating in-scanner head pose changes during structural MRI^16^.

Another approach to investigate the dynamics of data sequences is to apply models based on recurrent neural networks (RNN^17^). Figure 1c shows a time series data prepared for deployment of an LSTM model to solve the task described above. Since the data dependencies are captured by the ML algorithm, lagged variables are no longer necessary. Instead, demographic features are appended to the clinical features at each time step to form equal-length sequences and, by that, facilitate model training. RNNs have the advantage over non-temporal ML models in that they can maintain contextual information across the entire sequence. Nevertheless, vanilla RNNs are known for their vanishing gradient issues^18^ which make it difficult to discover latent patterns over a long sequence of data. Long Short-Term Memory (LSTM) networks^19^ are a variant of RNN designed to address this issue and have delivered promising success in the medical domain^20–23^. We provide a brief introduction to the LSTM framework in the “Materials and methods” section.

Our study focuses on comparing the effectiveness of the Differential and the LSTM approaches in predicting hospitalization for patients with SLE. The two techniques are fundamentally different: the former explores limited temporal dependencies in order to leverage a wide variety of non-temporal models, while the latter focuses on the full exploitation of temporal phenomena employing a narrow class of RNN models. Another key difference is the technique used to capture disease progression in longitudinal data. The Differential approach relies heavily on feature engineering in the pre-processing stage that occurs before model training. The LSTM model, on the other hand, draws its strength from the model’s particular architecture and the learning process applied to train it. Thus, our study is a comparison of two distinct approaches to model longitudinal EHR data. Our findings suggest that the Differential approach is more stable than the LSTM model. However, the LSTM model can achieve notably better performance under certain conditions.

In our motivating domain, the practitioners are interested in both long- and short-term prognoses of SLE patients to necessitate close monitoring and proper treatment administration. Thus, we formulated our machine learning task to predict a patient’s hospitalization at a future horizon (*Y*) using a period of clinical observations (*X*). We examined the two models’ performance over a set of varying *X* and *Y* time intervals and strive to identify the optimal method for each $X\xrightarrow{{predict}}Y$ task. An essential parameter for temporal machine learning models is the length *l* of the input sequences. In our study, *l* corresponds to the number of time intervals needed to collect the longitudinal clinical and lab data. While using an exceedingly small *l* could result in inadequate information to render an effective prediction, an overly large *l* will incur unnecessary costs and, more importantly, delayed treatment. Capitalizing on the experimental results, we also identified the optimal patient monitoring period (*X*) for each prediction horizon (*Y*).

## Materials and methods

### Data and preprocessing

We utilized our study on longitudinal EHR-based clinical records from the Massachusetts General Brigham (MGB) lupus study cohort, which includes patients from two large academic medical centers and multiple community hospitals. Our study population includes 925 patients identified from the MGB’s observational, EHR-based lupus cohort from 2016 to 2019. These patients were selected from a previous SLE phenotype study^24^ cohort, with the additional requirement of at least two visits with an MGB rheumatologist during the period of data accrual. Since each patient’s record forms a longitudinal time series with 6M intervals, we performed missing variable estimation for a given variable using linear interpolation/extrapolation fitted to its observed data points.

Each patient’s data includes five demographic and 52 time-stamped features that were selected by an SLE domain expert from readily available electronic health record data with clinical relevance to SLE. The latter is derived from categories including clinical manifestations, SLE medications, laboratory values, and healthcare utilization. Supplemental Table S1 provides the detailed features included in each category. This study was approved by the Mass General Brigham Institutional Review Board, and informed consent was waived. All procedures were carried out following relevant guidelines and regulations.

The outcome of interest was hospitalization for SLE hospitalization (defined as a primary SLE discharge diagnosis code of ICD-9 7.10.0 and ICD-10 M32.* excluding M32.0). We incorporated features using a period of clinical observations (*X*) to predict this outcome at a future horizon (*Y*). We denote such a task as:

$$X\xrightarrow{{predict}}Y$$

For *X*, we utilized with data sequences formed using time steps of 6, 12, 18, and 24 months, respectively. For *Y*, we varied the prediction horizons in 3, 6, 9, and 12 months, respectively. The above time intervals were provided by our domain experts based on their practical values.

The longitudinal patient records are irregular and unevenly distributed, owing to the nature of medical records. To form regular temporal sequences across all patients, we divided the data according to the desired observation interval *X* for each experiment. Multiple visits within a same time step were averaged to offer a set of observations equivalent to one clinical visit. Data averaging was applied only to lab features when a patient had multiple lab visits within the same time step. Per our domain expert’s recommendation, averaging the lab results within a 6M interval is acceptable for the chronic disease with the potential benefit of reducing the noise in the data.

### Differential approach

As illustrated in Fig. 1b, our Differential approach captures a patient’s disease progression by setting the longitudinal data to a time series structure and lagged at 6-month intervals to capture the disease progression. We assembled the model’s training data *D* for a given observation interval *X* and prediction period *Y* in two stages. First, independent training sequences of length *X* were extracted while moving step-wise along the time series. We labeled the instances with corresponding target outcome (i.e., with- or without-hospitalization) at time *Y* on the horizon. As an illustration, for the task of $2Y \xrightarrow {predict} 6M$, the goal was to use information in a 2-year observation interval to predict patients’ hospitalization outcomes in the next 6 months. The training data was created as follows for each patient $p_i$ $( i = 1, 2, \ldots , 925)$:

1 $$\begin{aligned} \begin{aligned} \{x^0, x^1, x^2, x^3\}_{p_i}&\xrightarrow {predict} y^{4}_{p_i}\\ \{x^1, x^2, x^3, x^4\}_{p_i}&\xrightarrow {predict} y^{5}_{p_i}\\ \{x^2, x^3, x^4, x^5\}_{p_i}&\xrightarrow {predict} y^{6}_{p_i}\\ &\vdots \\ \{x^{n-4}, x^{n-3}, x^{n-2}, x^{n-1}\}_{p_i}&\xrightarrow {predict} y^{n}_{p_i} \end{aligned} \end{aligned}$$

where $x^i$ denotes input time step *i* and each 2-year observation period consists of four time steps. *n* is the total length of the original time series. $y^i$ denotes the class label in 6 months intervals. The final dataset *D* consists of all sequences extracted from all patients. In the second stage, additional lagged variables were created between consecutive time steps for each training instance as illustrated in Fig. 1b.

The number of training sequences in *D* and their corresponding class labels are dependent on the choice of *X* and *Y* because fewer sequences can be extracted with a longer observation interval *X* and the prevalence of class 1 patients increases with larger *Y* because a longer prediction period leads to increased chances of hospitalization. Table 1 presents the distribution of *D*’s size and the number of class 1 (i.e., SLE hospitalization) instances across *X* and *Y* values selected for our study. In particular, the number of training instances decreases by 925 at each 6M increment of *X* (e.g., from 5550 to 4625 as *X* changes from 6M to 12M). This is because each patient will contribute exactly one less sequence when the observation interval is increased by one period.

**Table 1:** Data distribution across observation window *X* and prediction horizon *Y*.

*X*	*Y*							
	3M		6M		9M		12M	
	Total	Class 1	Total	Class 1	Total	Class 1	Total	Class 1
6M	5550	117 (2.11%)*	5550	207 (3.73%)	5550	286 (5.15%)	5550	358 (6.45%)
12M	4625	99 (2.14%)	4625	172 (3.72%)	4625	234 (5.06%)	4625	291 (6.29%)
18M	3700	76 (2.05%)	3700	131 (3.54%)	3700	181 (4.89%)	3700	221 (5.97%)
24M	2775	59 (2.13%)	2775	96 (3.46%)	2775	131 (4.72%)	2775	160 (5.77%)
30M	1850	36 (1.95%)	1850	59 (3.19%)	1850	74 (4.00%)	1850	96 (5.19%)
36M	925	12 (1.30%)	925	24 (2.59%)	925	31 (3.35%)	925	44 (4.76%)

*Number in parentheses indicates percentage of class 1 (SLE hospitalization) instances out of total. *X* and *Y* denote observation window and prediction horizons, respectively.

### LSTM model

**Figure 2:**
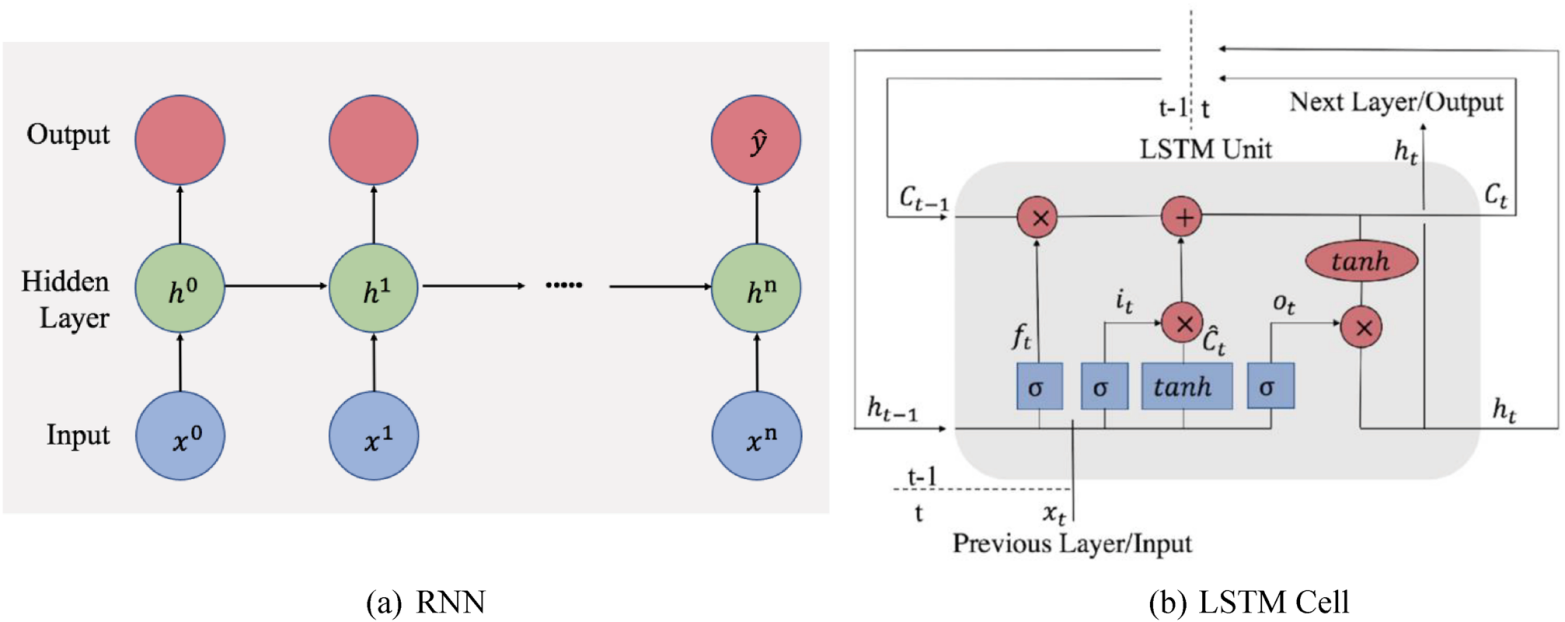
RNN and LSTM Cell. (**a**) Unfold illustration of RNN for training time series data of length *n*. Same model structure and weights are used for each time period. Adjacent time steps are connected via recurrent nodes in the hidden layer. Each subsequent step receives information from current input and previous hidden layer. Prediction is obtained at the last time step ($\hat{y}$). (**b**) Cell that replaces hidden nodes in (**a**) in the LSTM model.

Long short-term memory (LSTM)^19^ belongs to the family of recurrent neural networks (RNNs)^17^, which is designed to model sequential or time series data. As illustrated in Fig. 2a, an RNN architecture accounts for information from a contextual window of arbitrary length *n* via the edges that connect adjacent time steps. The same model structure and weights are used for each time period. Adjacent time steps are connected via recurrent nodes in the hidden layer. Prediction is obtained at the last time step ($\hat{y}$). LSTM is a variant of RNN developed to mitigate the vanishing gradient problem that can be encountered when training traditional RNNs^18^. As illustrated in Fig. 2b, the LSTM model augments the traditional RNN hidden nodes with a memory cell. Inside each cell, three “regulators” help LSTM selectively remember and forget information passed into the cell. These regulators are named input gate, output gate, and forget gate. Specifically,

- Input gate: this gate decides what information is relevant to add to the cell for the current step. It takes activation from the current time step as well as from the hidden layer at the previous time step. If the gate’s value is zero, the flow from another node is cut off, whereas if its value is one, all flow is passed, i.e.,
  2 $$\begin{aligned} i_t = \sigma (W_i \dot{[}h_{t-1}, x_t] + b_i) \end{aligned}$$
- Forget gate: this gate regulates what information to discard from the cell. This decision is made by a sigmoid layer applied to the previous hidden state $h_{t-1}$ and current input $x_t$, i.e.,
  3 $$\begin{aligned} f_t = \sigma (W_f \dot{[}h_{t-1}, x_t] + b_f) \end{aligned}$$
- Output gate: this gate decides what the next hidden state should be. Similar to the forget gate, it is another sigmoid layer applied to the previous hidden state $h_{t-1}$ and current input $x_t$, i.e.,
  4 $$\begin{aligned} o_t = \sigma (W_o \dot{[}h_{t-1}, x_t] + b_o) \end{aligned}$$
- Finally, an updated cell state ($C_t$) and a new hidden state ($h_t$) will be passed to the next cell as follows:
  5 $$\begin{aligned} C_t = f_t \circ C_{t-1} + i_t \circ \widetilde{C_t} \end{aligned}$$
  $$h_t = o_t \circ \tanh (C_t)$$
  where $\widetilde{C_t} = \tanh (W_c \dot{[}h_{t-1}, x_t] + b_c])$ and operator $\circ$ denotes element-wise multiplication.

The *W*’s and *b*’s in Eqs. (2)–(5) denote the weight matrices and bias vectors that need to be learned during training. Our training data for the LSTM model is the same as the Differential approach in terms of size and positive instance prevalence. However, the LSTM data does not include the extra lagged variables because the temporal dependencies of the time series are captured by the model architecture.

We assembled the LSTM model’s training data following the same two-stage process outlined in for the Differential approach. In the first stage, independent training sequences were extracted according to Eq. (1) depending on the observation interval *X* and prediction period *Y*. In the second stage, equal-length time series data was formed using the process described in Fig. 1c.

### Addressing data imbalance

We observe in Table 1 that the prevalence of SLE hospitalization events (class 1) ranges from 1.3 to 6.45% in the study data depending on the *X* and *Y* values. This severe imbalance in class distribution poses a challenge for predictive modeling because standard machine learning algorithms assume an equal number of class representations in the training data. Learning directly from an imbalanced dataset would lead to unsatisfactory performance in the minority class when the algorithms strive to minimize a global loss. While there are various techniques to rectify the data imbalance issue in training ML models, some methods such as undersampling and SMOTE^25^ are ineffective for severely imbalanced datasets due to their technical limitations^26^.

For the Differential approach, we experimented with the oversampling and the bootstrap aggregating with random undersampling^27^ (i.e., bagging) methods, and the latter led to better performance with consistent higher AUC scores across various observation (*X*) and prediction (*Y*) intervals. Detailed comparison results are provided in Supplemental Fig. S2. We used the better model to compare with the LSTM approach. In particular, the “bagging” technique learns multiple decision boundaries between the minority and various subsets of majority samples and simultaneously leverages the advantages of ensemble learning. To this end, we first generated 100 “bags” of balanced datasets from the training data, where each “bag” contained all minority instances and an equal number of majority instances randomly sampled (with replacement) from the entire majority population. We next trained 100 sub-models on the balanced “bags” and aggregated the results of all sub-models by averaging their class predictive probabilities. The number of bags (i.e., 100) was selected as a hyperparameter. For the LSTM model, due to its computational constraints, we addressed the class imbalance issue by oversampling^28^. That is, each minority sequence is duplicated $r-1$ times to create balanced training data, where *r* is the ratio between the majority and minority instances.

### Experimental framework

**Figure 3:**
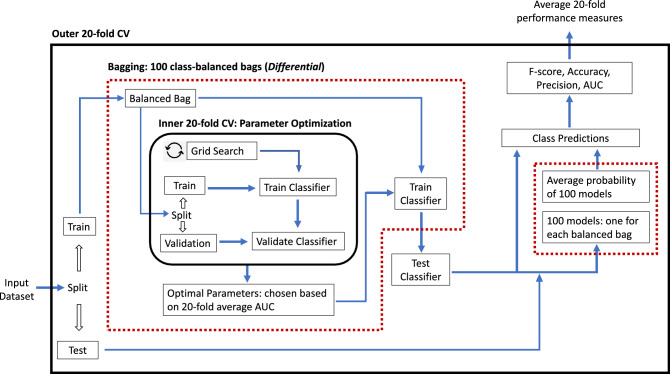
Model training framework. Red boxes indicate additional layers in the Differential approach due to the bagging method in addressing class imbalance.

Figure 3 illustrates our model training framework. We evaluated each model’s performance using a 20-fold (outer) cross-validation. The process involves randomly splitting the entire dataset into 20 disjoint groups (i.e., folds), of approximately equal size. Subsequently, each model is trained 20 times using the $i$-th ($i=1, 2, \ldots , 20$) fold as the test data, and the remaining 19 folds as the training data ($T_i$). For each evaluation metric, we report a model’s performance as the mean of the 20 out-of-sample scores on the 20 test folds, indicated by the upper right box in Fig. 3.

Red boxes in Fig. 3 indicate additional layers for the Differential approach corresponding to the bagging method used in addressing class imbalance. Therein, each Differential’s training iteration produces 100 sub-models fitted on 100 balanced bags. We compute the class label of each test instance by averaging its class scores produced by the 100 sub-models. For the LSTM models, since we replaced bagging with oversampling, each of its training iterations produces one test classifier trained on a balanced dataset with duplicated minority instances.

Lastly, we applied a nested cross-validation to facilitate hyper-parameter selection. To this end, we further partitioned training data $T_i$ in each outer iteration *i* ($i=1, 2, \ldots , 20$) into 20 folds and conduct a grid search^29^ on a set of algorithm-specific parameters. The optimal parameter set ($P_i$) for $T_i$ was chosen to produce the highest average AUC (Area Under the ROC Curve)^30^ score on the 20 test folds. We reported the performance of the model trained using $T_i$ and $P_i$.

We trained our models on a PowerEdge R740 Linux machine with two Xeon 2.60GHz CPUs (12 cores), 192GB of memory, and a 32GB NVIDIA Tesla V100 GPU. We trained the LSTM model for 50 epochs with a batch size of 72. The convergence was accomplished using the Adam optimizer with a learning rate of 0.0001, minimizing the cross-entropy loss between the model output and class labels.

We evaluated the Differential approach’s performance using a majority-voting ensemble of six non-temporal base learners, namely, decision trees^10^, random forests^11^, logistic regression^12^, naive Bayes, neural network^13^, and support vector machine^31^. In addition to overall accuracy, we compared recall and specificity to study the models’ respective efficacy in the positive and negative classes across varying observation periods (i.e., *X* = 6M, 12M, 18M, 24M, 30M, and 36M) and varying prediction horizons (i.e., *Y* = 3M, 6M, 9M, and 12M). We further compared the models using additional evaluation metrics, including AUC, PPV, F1, and F0.

## Results

**Figure 4:**
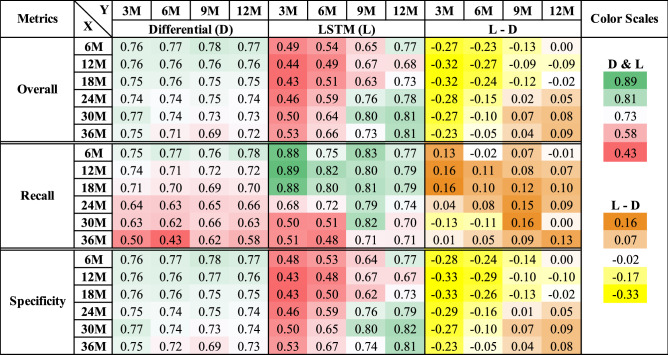
Performance comparison in overall accuracy, recall, and specificity. Each row represents an observation period *X*, and each column represents a prediction horizon *Y*. Performance blocks under the “Differential” and “LSTM” columns adopt color scale green-to-red for values from high to low. Blocks under the “L–D” column uses a brown-to-yellow scale indicating large to small gains LSTM has over Differential.

Figure 4 displays the overall accuracy, recall, and specificity across varying feature assessment periods and outcome assessment periods. We applied color scales to visualize the relative performance. The color scale is green-to-red for the performance blocks under the “Differential” and “LSTM” columns, corresponding to values from high to low. Blocks under the “L–D” column uses a brown-to-yellow scale indicating large to small (can be negative) gains LSTM has over Differential.

### Overall trends

From the overall accuracy blocks in Fig. 4 (Row #1), we observe that the Differential approach is stable compared to the LSTM model. We believe this is due to the bagging technique, which not only balanced the training data but also offers the benefit of ensemble learning^32^ (i.e., an ensemble of 100 sub-models). Although it was infeasible to institute the same method for LSTM due to resource limitations, LSTM achieved notably better performance than Differential with longer intervals of the feature assessment period *X* and longer intervals of the outcome assessment period *Y* (i.e., the lower right regions). One explanation for LSTM’s advantage in this region is the long observation periods and the model’s efficacy in capturing long-term contextual information in the time series data.

Another observation is that LSTM’s predictions are generally biased towards the positive class. This pattern is evidenced by the brown-colored cells in the “L–D” block for the Recall metric and the yellow-colored cells for the respective Specificity metric. A potential explanation for LSTM’s discriminatory behavior is due to the oversampling technique, which is equivalent to imposing extra penalties when the model misclassifies positive instances. We discuss this limitation in more detail in the “Discussion” section.

### Performance analysis across *X* values

Intuitively, longer monitoring intervals (*X*) are likely to bring performance gains due to extra clinical information. However, a larger *X* value also leads to a smaller size of training data (Table 1). Thus, there is a trade-off between the length of the monitoring window and the number of training samples. We observe that the Differential approach’s overall performance decreases as the monitoring window *X* increases for all predictive horizons (*Y*), suggesting that the reduction in sample size outweighs the extra information embedded in the data. The LSTM model exhibits the same pattern when *Y* = 3M and 6M. However, the trend reverses with $Y>$ 6M where the LSTM’s performance increases alongside the *X* values. This performance gain is likely due to the increased number of positive training samples, which helped the LSTM capture long-term dependencies in the sequential data.

In addition, the Differential results are most effective with short (*X* = 6M and 12M) observation intervals. The approach loses its efficacy as *X* increases. This trend is particularly pronounced for class 1 (Recall blocks), where the accuracy is above 88% for $X<18$M and dropped to approximately 50% with a 36M observation. On the contrary, the LSTM model is more effective with larger *X* values, further confirming LSTM’s ability to capture long-term contextual information in the data. For instance, LSTM’s recall accuracy achieved 74% when $X=24$M, but the Differential approach’s performance was 64%. A similar pattern exists for Specificity. For $X=24$M, the Specificity for LSTM and Differential results are 79% and 74%, respectively.

### Performance analysis across *Y* values

We expect a model’s overall performance to improve as the predictive horizon *Y* increases because the raised percentage of positive (class 1) instances alleviates the class imbalance issue. This pattern is salient for LSTM by examining the overall accuracy metric blocks, where LSTM’s best overall performance improved from 53% to 81% across different *Y* values. In contrast, the Differential approach maintained a stable performance across all *Y* values, which indicates that the bagging technique can address highly imbalanced datasets. Based on the results in the “L–D” blocks, LSTM starts to outperform Differential when the prediction horizon *Y* is longer than 6M. Together with the above findings over the *X* values, our study suggests employing the LSTM model when $Y>$ 6M and $X>$ 18M and the Differential approach for the remaining cases.

### Comparison on additional performance metrics

**Figure 5:**
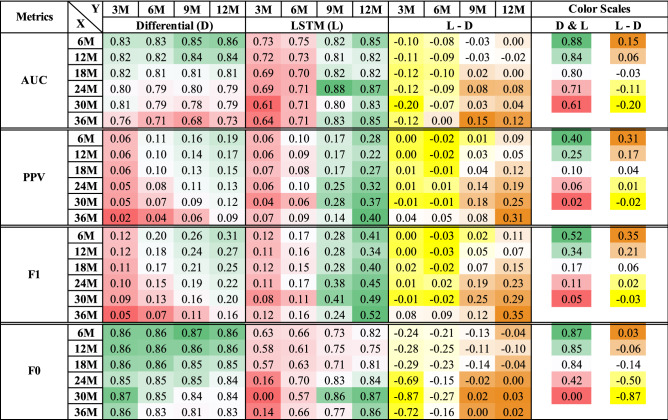
Comparisons on additional evaluation metrics.

Figure 5 presents comparisons on additional evaluation metrics (AUC, PPV, F1, and F0) between the Differential and LSTM approaches. Each column follows the same color schedule as in Fig. 4, that is, green-to-red for Columns “D” and “L” and brown-to-yellow for Column “L–D”. We further adjusted the color scales according to the min/max values of each evaluation metric to account for different value ranges. All blocks under the “L–D” column consistently indicate that the gain of LSTM over Differential is evident when moving towards longer durations for X and Y. The Differential approach had a maximum AUC of 0.86 for a 6-month feature assessment period and a 12-month outcome assessment period. The LSTM model achieved a maximum AUC of 0.88 for 24 months of feature assessment and 9 months of outcome assessment. For the PPV measure, the Differential approach’s precision decreased with large *X* values. The highest precision for class 1 (0.19) was achieved at *X* = 6M and *Y* = 12M. Conversely, the LSTM model’s precision improved with larger *X* values and achieved its highest PPV value of 0.52 at *X* = 36M and $Y=12$M. This opposite trend between the two models once again highlights LSTM’s strength in modeling long-term data dependencies, which is further confirmed by the same patterns in the F1 and F0 measures.

### Optimal observation windows

To identify the best *X* value for each *Y* target, we focused on the AUC scores in Fig. 5 and followed the model selection analysis across the *X* and *Y* values. Specifically, for $Y=9$M and 12M, the optimal choice is $X= 24$M (i.e., a 2-year total observation). The respective AUC scores are 0.87 and 0.88, achieved by the LSTM model. For $Y=3$M and 6M, the Differential results showed higher AUC scores, which decrease as X increases. Thus, for a short predictive horizon, it is sufficient to monitor the patient for a period of 6M using the non-temporal models with a Differential approach.

## Discussion

We aimed to predict the likelihood of hospitalization for SLE in the next 3–12 months, within a multicenter EHR-based SLE cohort. The task is essential in managing patients’ risks of developing irreversible organ damage^33,34^ and lower health-related quality of life^35^, and also impacts the direct cost of SLE care^2^ but is challenging given the heterogeneous nature of this disease with variability in disease course^1^. We utilized longitudinal EHR data and explored two temporal ML models to capture disease course development in the time series data. The Differential approach accounts for the temporal dependencies by introducing additional lagged variables between consecutive time steps. As demonstrated in this study, the technique can be adopted by any non-temporal machine learning methods that assume all features are independent. The LSTM approach capitalizes on the model’s architecture to memorize sequential data’s contextual information.

To accommodate different clinical needs, we experimented with predicting a patient’s hospitalization at various future horizons (*Y*) using different observation windows (*X*). Our findings suggest that LSTM outperforms the Differential approach only when *X* is sufficiently large. Since a larger *X* implies longer training sequences, the results are consistent with LSTM’s reputation in retaining long-term contextual information in sequential data.

Our findings further suggest that LSTM outperforms the Differential approach only when *Y* is sufficiently large. Since large *Y* values are associated with more positive training samples, one explanation for this outcome is the limited availability of positive instances and the oversampling technique employed to handle the imbalanced data. In particular, for a highly imbalanced dataset, bagging can be preferred in addressing class imbalance because it learns multiple decision boundaries between the minority and various subsets of majority samples and simultaneously leverages the advantages of ensemble learning. Due to the time constraints in training LSTM models, we replaced bagging with oversampling, which is equivalent to increasing the misclassification penalty of the minority class by $r=\frac{1}{p}$ times, where *p* is the percentage of minority samples. Consequently, for $Y=3$M, low *p* values (1.3–2.14%, Table 1) led to excessive *r* values, resulting in LSTM’s biased predictions of positive samples. As the interval for *Y* increases, LSTM becomes more effective as *r* decreases. While it is tempting to search for an optimal *r* as a model hyper-parameter, the exploration would again lead to computational resource constraints. Since LSTM demands a higher balance of the underlying data, we recommend training an LSTM model for predicting SLE patients’ hospitalization only if the interested prognostic interval is above 6 months.

Lastly, we recognize two limitations in our study. First, the EHR data were collected as part of clinical care and not for primary research purposes. As a result, clinical visits and lab results are captured at irregular intervals. Furthermore, sicker patients may have more frequent visits than healthier patients, resulting in a higher volume of patient information. Second, the hospitalization outcomes are limited to the MGB healthcare system. Thus, our data may contain misclassified labels for patients hospitalized outside of the system. However, due to the large sample size and a prior validated SLE phenotype study for these patients^24^, we believe the misclassification bias is minimal in our cohort.

## Conclusion

Our study compares the efficacy of two temporal machine learning approaches in predicting SLE hospitalizations using EHR data. Our experimental results demonstrate that both methods can be effective for our task but each has its strengths and limitations. The Differential approach can be integrated into all non-temporal machine learning algorithms and is suitable for short observation periods. Conversely, LSTM excels at capturing long-term dependencies embedded in the longitudinal data and, thus, is desirable for tasks with long-term observation windows. In addition, the Differential approach is adept in handling class imbalance in model training and delivers stable performance across different prognostic intervals whereas LSTM demands a higher quality of the underlying data and outperforms Differential when there are sufficient positive samples facilitating model training. These models can be applied accordingly to predict future SLE hospitalizations from various patient monitoring periods ranging from 6M to 36M. We further suggest 6M and 24M as the desirable observation windows for short- and long-term assessment horizons, respectively. Our approach could be applied to other clinical conditions to leverage time-dependent EHR data to predict longitudinal health outcomes.

## Supplementary Information

Supplementary Table S1.

Supplementary Figure S2.

## Data Availability

The data used in the current study is not publicly available due its proprietary nature but will be provided to qualified investigators upon reasonable request. Requests should be directed to Dr. April Jorge, AMJORGE@mgh.harvard.edu.
