# Supplementary material for: Comparing two machine learning approaches in predicting lupus hospitalization using longitudinal data
Source: Sci Rep. 2022 Sep 30;12:16424. doi: 10.1038/s41598-022-20845-w (PMC9525268; doi:10.1038/s41598-022-20845-w)
Supplement: Supplementary file 1 — Supplementary Table S1. [file 41598_2022_20845_MOESM1_ESM.pdf]

Table S1. Features and Data Source

Feature	Data Source
**Demographics**	
Age	Structured fields
Sex	Structured fields
Race	Structured fields
Hispanic ethnicity	Structured fields
Marital status	Structured fields
**Socioeconomic Factors**	
Insurance payor	Structured fields
**Clinical Manifestations**	
Nephritis	ICD-9 580.*, 583.*, N05.*, N03.2*, 791.0, R80.*; ICD 10 M32.14, M32.15
Pericarditis	ICD-9 420.*; ICD-10 I30.*, I32.*, I31.*, M32.12
Pleurisy	ICD-9 511.9, 511.0, 511.89; ICD-19 R09.1
Myocarditis	ICD-9 429.0, 422.0, 422.9; ICD-10 I40.8, I40.9, I51.4,
Hemolytic anemia	ICD-9 283.*; ICD-10 D59.*
Cutaneous lupus	ICD-9 695.4; ICD-10 L93.0, L93.1, L93.2,
Vasculitis	ICD-9 447.6; ICD-10 I77.6, M31.8,
Demyelinating syndrome or transverse myelitis	ICD-10 G37.3, G04.89
Acute Renal impairment	ICD-9 584; ICD-10 N17.*
**SLE Medications**	
Oral corticosteroids	Prescriptions
Hydroxychloroquine	Prescriptions
Azathioprine	Prescriptions
Methotrexate	Prescriptions
Mycophenolate	Prescriptions
Belimumab	Prescriptions
Cyclophosphamide	Prescriptions
Rituximab	Prescriptions
Other (incl. tacrolimus, cyclosporine, leflunomide, abatacept)	Prescriptions
**Laboratory Test Values**	
White blood cell count	Average value
Hemoglobin/hematocrit	Average value
Platelet count	Average value
Creatinine	Average value
AST	Average value
ALT	Average value
Albumin	Average value
Erythrocyte sedimentation rate	Average value
C-reactive protein	Average value
Urine protein	Average value
Urine protein/creatinine ratio	Average value
Urine red blood cells	Average value
C3	Average value
C4	Average value
ANA titer	Average value
ANA pattern	Mode
B2 glycoprotein 1 antibody IgG	Frequency positive
B2 glycoprotein 1 antibody IgM	Frequency positive
Anti-dsDNA antibody	Frequency positive
Anti-smith antibody	Frequency positive
Anti-ribonucleoprotein	Frequency positive
Anti-Sjogren's syndrome A	Frequency positive
Anti-Sjogren's syndrome B	Frequency positive
**Healthcare utilization**	
Rheumatology visits	Counts of encounters
Dermatology visits	Counts of encounters
Nephrology visits	Counts of encounters
Other clinical visits	Counts of encounters
