# Supplementary material for: Comparing two machine learning approaches in predicting lupus hospitalization using longitudinal data
Source: Sci Rep. 2022 Sep 30;12:16424. doi: 10.1038/s41598-022-20845-w (PMC9525268; doi:10.1038/s41598-022-20845-w)
Supplement: Supplementary file 2 — Supplementary Figure S2. [file 41598_2022_20845_MOESM2_ESM.pdf]

Metrics	X \ Y	3M	6M	9M	12M	3M	6M	9M	12M	3M	6M	9M	12M	Color Scales
		Bagging (B)				Oversampling (O)				O - B				
Overall	6M	0.76	0.77	0.78	0.77	0.76	0.74	0.73	0.74	0.00	-0.03	-0.04	-0.03	
	12M	0.76	0.76	0.76	0.76	0.73	0.71	0.74	0.69	-0.03	-0.05	-0.03	-0.07	B & O
	18M	0.75	0.76	0.75	0.75	0.75	0.71	0.72	0.70	-0.01	-0.05	-0.03	-0.05	0.90
	24M	0.74	0.74	0.75	0.74	0.71	0.71	0.71	0.72	-0.04	-0.03	-0.04	-0.02	0.82
	30M	0.77	0.74	0.73	0.73	0.76	0.76	0.75	0.73	0.00	0.02	0.03	0.00	0.73
	36M	0.75	0.71	0.69	0.72	0.89	0.79	0.70	0.78	0.14	0.08	0.01	0.06	0.50
Recall	6M	0.75	0.77	0.76	0.78	0.62	0.67	0.72	0.73	-0.13	-0.09	-0.04	-0.04	0.26
	12M	0.74	0.71	0.72	0.72	0.65	0.69	0.68	0.73	-0.08	-0.02	-0.04	0.01	
	18M	0.71	0.70	0.69	0.70	0.56	0.65	0.67	0.68	-0.15	-0.05	-0.01	-0.01	
	24M	0.64	0.63	0.65	0.66	0.58	0.56	0.62	0.61	-0.06	-0.07	-0.03	-0.05	O - B
	30M	0.63	0.62	0.66	0.63	0.52	0.47	0.51	0.55	-0.11	-0.15	-0.15	0.00	0.15
	36M	0.50	0.43	0.62	0.58	0.28	0.26	0.44	0.34	-0.22	-0.17	-0.18	-0.24	0.06
Specificity	6M	0.76	0.77	0.78	0.77	0.76	0.74	0.73	0.74	0.00	-0.02	-0.05	-0.03	-0.03
	12M	0.76	0.76	0.77	0.76	0.73	0.71	0.74	0.68	-0.03	-0.05	-0.03	-0.08	-0.14
	18M	0.76	0.76	0.75	0.75	0.75	0.71	0.72	0.70	-0.01	-0.05	-0.03	-0.05	-0.24
	24M	0.75	0.74	0.75	0.74	0.71	0.72	0.71	0.72	-0.04	-0.02	-0.04	-0.02	
	30M	0.77	0.74	0.73	0.74	0.77	0.76	0.76	0.74	0.00	0.02	0.03	0.01	
	36M	0.75	0.72	0.69	0.73	0.90	0.80	0.70	0.81	0.15	0.08	0.01	0.08	

Metrics	X \ Y	3M	6M	9M	12M	3M	6M	9M	12M	3M	6M	9M	12M	Color Scales	
		Bagging (B)				Oversampling (O)				O - B				B & O	O - B
AUC	6M	0.83	0.83	0.85	0.86	0.74	0.76	0.79	0.81	-0.09	-0.07	-0.06	-0.04	0.86	-0.04
	12M	0.82	0.82	0.84	0.84	0.74	0.76	0.77	0.78	-0.08	-0.06	-0.07	-0.06	0.81	-0.06
	18M	0.82	0.81	0.81	0.81	0.72	0.75	0.76	0.76	-0.09	-0.05	-0.05	-0.05	0.77	-0.08
	24M	0.80	0.79	0.80	0.79	0.72	0.69	0.73	0.72	-0.08	-0.10	-0.07	-0.07	0.67	-0.11
	30M	0.81	0.79	0.78	0.79	0.72	0.68	0.70	0.71	-0.09	-0.11	-0.08	-0.08	0.58	-0.14
	36M	0.76	0.71	0.68	0.73	0.67	0.58	0.58	0.59	-0.09	-0.13	-0.10	-0.14		
PPV	6M	0.06	0.11	0.16	0.19	0.06	0.10	0.15	0.20	-0.01	-0.01	0.00	0.00	0.20	0.01
	12M	0.06	0.10	0.14	0.17	0.05	0.09	0.14	0.14	-0.01	-0.02	0.00	-0.03	0.14	0.00
	18M	0.06	0.10	0.13	0.15	0.05	0.08	0.12	0.14	-0.01	-0.01	-0.01	-0.01	0.09	-0.01
	24M	0.05	0.08	0.11	0.13	0.05	0.07	0.11	0.13	-0.01	-0.01	-0.01	-0.01	0.06	-0.02
	30M	0.05	0.07	0.09	0.12	0.05	0.07	0.09	0.11	0.00	0.00	0.00	0.00	0.02	-0.03
	36M	0.02	0.04	0.06	0.09	0.03	0.03	0.05	0.08	0.01	-0.01	-0.01	-0.02		
F1	6M	0.12	0.20	0.26	0.31	0.10	0.17	0.24	0.29	-0.02	-0.03	-0.02	-0.02	0.31	0.01
	12M	0.12	0.18	0.24	0.27	0.10	0.15	0.22	0.23	-0.02	-0.03	-0.02	-0.04	0.23	0.00
	18M	0.11	0.17	0.21	0.25	0.08	0.14	0.20	0.23	-0.02	-0.02	-0.01	-0.02	0.16	-0.02
	24M	0.10	0.15	0.19	0.22	0.08	0.12	0.18	0.20	-0.01	-0.03	-0.02	-0.02	0.10	-0.03
	30M	0.09	0.13	0.16	0.20	0.08	0.11	0.15	0.18	-0.01	-0.02	-0.02	-0.02	0.05	-0.05
	36M	0.05	0.07	0.11	0.16	0.06	0.05	0.09	0.11	0.01	-0.02	-0.03	-0.05		
F0	6M	0.86	0.86	0.87	0.86	0.86	0.84	0.83	0.84	-0.01	-0.02	-0.04	-0.03	0.94	0.08
	12M	0.86	0.86	0.86	0.86	0.84	0.82	0.84	0.80	-0.02	-0.04	-0.02	-0.06	0.89	0.03
	18M	0.86	0.86	0.85	0.85	0.85	0.82	0.83	0.81	-0.01	-0.04	-0.02	-0.04	0.85	-0.02
	24M	0.85	0.85	0.85	0.84	0.81	0.82	0.81	0.82	-0.04	-0.03	-0.04	-0.02	0.81	-0.04
	30M	0.87	0.85	0.84	0.84	0.86	0.85	0.85	0.83	-0.01	0.01	0.01	-0.01	0.78	-0.06
	36M	0.86	0.83	0.81	0.83	0.94	0.87	0.78	0.87	0.08	0.05	0.00	0.04		

Figure S2. Comparisons of Bagging and Oversampling in Addressing Data Imbalance for the Differential Approach. Each row represents an observation period X, and each column represents a prediction horizon Y. Performance blocks under the "Bagging (B)" and "Oversampling (O)" columns adopt color scale green-to-red for values from high to low. Blocks under the "O - B" column uses a brown-to-yellow scale indicating the differences between bagging and oversampling methods.
